# MiR‐133a acts as a tumor suppressor in lung cancer progression by regulating the LASP1 and TGF‐**β**/Smad3 signaling pathway

**DOI:** 10.1111/1759-7714.13678

**Published:** 2020-10-19

**Authors:** Yuyao Shen, Yan Yang, Yahua Li

**Affiliations:** ^1^ Department of Respiratory Medicine The Affiliated Yantai Yuhuangding Hospital of Qingdao University Yantai China; ^2^ Department of Respiratory Medicine Shan Dong Chest Hospital Jinan China; ^3^ Department of Respiratory Medicine The Third Hospital of Hebei Medical University Shijiazhuang China

**Keywords:** LASP1, miR‐133a, non‐small cell lung cancer, TGF‐β

## Abstract

**Background:**

MiR‐133a has been confirmed to be involved in the development of multiple cancers including non‐small cell lung cancer (NSCLC). However, the precise molecular mechanism has not yet been fully elucidated. The purpose of this study was to investigate the functional role and underlying mechanism of miR‐133a in the progression of NSCLC.

**Methods:**

Quantitative real‐time PCR (qRT‐PCR) was performed to measure miR‐133a and LASP1 expression in NSCLC tissues and cells. 3‐(4, 5‐dimethyl‐2‐thiazolyl)‐2, 5‐diphenyl‐2H‐tetrazolium bromide (MTT) assay was used to detect cell viability. The protein levels were measured by western blot. The tumor growth was measured by xenograft tumor formation assay.

**Results:**

miR‐133a was significantly decreased while LASP1 was increased in NSCLC tissues and cells compared with control groups. Moreover, overexpression of miR‐133a suppressed cell viability, whereas miR‐133a knockdown enhanced the viability of A549 cells. More importantly, LASP1 was verified as a direct target of miR‐133a. Moreover, overexpression of miR‐133a inhibited the epithelial‐mesenchymal transition (EMT) and TGF‐β/Smad3 pathways by regulating LASP1 in vitro. In addition, miR‐133a mimic suppressed tumor growth by modulating the TGF‐β/Smad3 pathway in vivo.

**Conclusions:**

In conclusion, miR‐133a acted as a tumor suppressor in lung cancer progression by regulating the LASP1 and TGF‐β/Smad3 signaling pathway.

## Graphical abstract

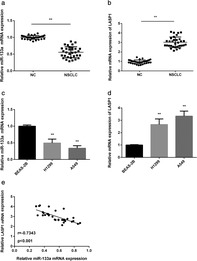

miR‐133a mimic suppressed cell viability and miR‐133a inhibitor enhanced the viability of A549 cells. LASP1 was verified as a direct target of miR‐133a. Overexpression of miR‐133a inhibited the EMT and TGF‐ß/Smad3 pathway by regulating LASP.

## Introduction

Lung cancer is the most commonly diagnosed cancer and the leading cause of cancer mortality worldwide. Non‐small cell lung cancer (NSCLC) accounts for nearly 85% of lung cancer cases, ranking first in cancer deaths worldwide.^1^ Despite significant advances in diagnosis and treatment, the overall five‐year survival rates are still very poor.^2^ Therefore, an understanding of the molecular mechanisms involved in the progression of NSCLC to develop new and effective treatment strategies is urgently required.

Clinically, tumor growth and metastasis are the leading cause of death in cancer patients, but, as yet, there are currently no effective treatment strategies.^3^ Epithelial‐mesenchymal transition (EMT) has been verified as an initiating mechanism of tumor metastasis and a complex process of embryonic development.^4^ Moreover, EMT allows tumor cells to infiltrate and metastasize to distant sites in the malignant progression of tumors, which is the basis of the development and occurrence of malignant tumors.^5^

MicroRNAs are highly conserved non‐coding RNA molecules that bind primarily to sequences in the 3′UTR and function as transcriptional functions by binding to mRNA. Previous studies have shown that miRNAs are closely correlated with diverse tumor development, including regulating colorectal cancer cell proliferation,^6^ cervical cancer cell migration and invasion,^7^ nasopharyngeal carcinoma cell proliferation and invasion^8^ and lung cancer cell proliferation and metastasis.^9^ There is increasing evidence that miR‐133a is closely associated with various cancers, such as prostate cancer,^10^ hepatocellular carcinoma^11^ and ovarian cancer.^12^ Wang *et al*. reported that miR‐133a was downregulated in NSCLC and associated with poor prognosis in patients.^13^ However, the significance of the role of miR‐133a in NSCLC tumor growth has not yet been fully clarified.

LIM and SH3 domain protein 1, also known as LASP1, was first reported by Tomasetto *et al*. in 1995.^14^ Previous studies have stated that LASP1 was increased significantly in multiple malignant tumors and is involved in the development of cancers. LASP1 has also been shown to be a target of signaling pathways, including the TGF‐β/Smad3 pathway.^15^, ^16^ Wang *et al*. discovered that LASP1 was significantly increased by stimulating colorectal cancer cells with the EMT inducer TGF‐β.^16^ Moreover, LASP1 has been reported to induce the EMT process and create aggressive cancer cell phenotypes, which thereby promotes cancer cell growth and metastasis.^17^ Fahrmanna *et al*. reported that LASP1 is associated with poor prognosis in lung cancer patients.^18^ Zheng *et al*. demonstrated that LASP1 enhanced NSCLC cell proliferation and aggressiveness related to the survival of lung cancer patients.^19^ However, the mechanism of LASP1 in NSCLC cell growth has not yet been fully elucidated.

In this study, we aimed to explore the role and mechanism of miR‐133a in modulating NSCLC cell proliferation and tumor growth. We found that miR‐133a was downregulated in NSCLC and showed an inhibitory effect on cell viability and tumor growth. Moreover, LASP1 was verified as a specific target of miR‐133a. In conclusion, miR‐133a suppressed cell progression via regulating the LASP1 and TGF‐β/Smad3 signaling pathway, providing a novel target for the treatment of NSCLC.

## Methods

### Sample collection and cell culture

A total of 32 specimens of NSCLC tissues and parallel normal tissues were obtained from the Third Hospital of Hebei Medical University between March 2010 and July 2012. All clinical protocols were approved by the Ethics Committees of the Third Hospital of Hebei Medical University (20130506) and patients signed their informed consent to be included in the study. Histological diagnosis and grading were evaluated according to the 2015 World Health Organization (WHO) classification of tumors of the lung.^20^ The 32 specimens were classified according to histological subtype, differentiation, and tumor stage. Tumor staging was performed according to the seventh edition of the International Union against Cancer (UICC) TNM Staging System for Lung Cancer.^21^ The median age of 32 patients was 56 years old (range 29–79 years old). RPMI‐1640 medium containing 10% FBS, purchased from the American type culture collection (ATCC), was used for maintaining NSCLC cells (A549, H1299) and human normal lung epithelial cells (BEAS‐2B). The cells were then incubated at 37°C in a humidified chamber at 5% CO_2_.

### RNA isolation and quantitative real‐time PCR

Total RNA was extracted or isolated from cells or tissues by Trizol reagent (Takara, China) according to the manufacturer's protocol. A nanodrop ultraviolet spectrophotometer was used to quantify and assess mRNA. A reverse transcription kit (Takara, China) was used to perform reverse transcription of RNA. SYBR Premix Ex TaqTM was applied to perform real‐time PCR. PCR primer for miR‐133a, forward, 5′‐TGCTTTGCTAGAGCTGGTAAAATG‐3′ and reverse, 5′‐AGCTACAGCTGGTTGAAGGG‐3′. The endogenous reference genes use U6, forward, 5′‐CTCGCTTCGGCAGCACA‐3′ and reverse, 5′‐AACGCTTCATTTGCGT‐3′. 5′‐GAGAGGAACAAGCTGGCTGC‐3′ and 5′‐GCTTCTCCTTCTCCTTCTGC ‐3′ for LASP1; The LASP1 expression level was normalized to GAPDH, forward, 5′‐CGGAGTCAACGGATTTGGTCGTAT‐3′; reverse, 5′‐AGCCTTCTCCATGGTGGTGAAGAC‐3′. All experiments were independently conducted three times and the ΔΔCt method was used to calculate gene expression.

### Cell transfection

MiR‐133a mimic, inhibitor or corresponding negative control was purchased from GenePharma (Shanghai, China). A549 cells were transfected with viral suspension when cells were in the logarithmic phase using Lipofectamine 2000 according to the manufacturer's protocol. After incubation for 48 hours, the transfection efficiency of miR‐133a was observed by qRT‐PCR. MiR‐133a mimic and negative control in A549 cells were stimulated with TGF‐β for 24 hours.

### Western blot

Lysis buffer was used to resolve the transfected cells, which were then centrifuged for 15 minutes at 4°C, 12000 rpm. A total of 50 μg proteins were added to 8%–15% polyacrylamide gels and then transferred to polyvinylidene fluoride (PVDF) membranes. After blocking with 5%–10% nonfat milk at room temperature for two hours with gentle agitation, the membranes were incubated with primary antibodies: anti‐GAPDH (1:1000, CST), anti‐LASP1 (1:500, ab1301, abcam), anti‐TGF‐β (1:500, ab92486, ab cam), anti‐E‐cadherin (1:1000, ab133597, abcam), anti‐N‐cadherin (1:1000, ab98952, abcam), anti‐Vimentin (1:500, ab20346, abcam), anti‐p‐Smad3 (1:2000, ab52903, abcam), and anti‐Smad3 antibody (1:1000, ab40854, abcam) at 4°C overnight. Subsequently, the corresponding secondary antibodies (1:2000, sc2030, Santa Cruz) were added to incubate the membranes for two hours at room temperature. They were then washed three times, and an enhanced chemiluminescence kit was used to detect the blots. GAPDH staining was used for normalization.

### MTT assay

MTT assay was used to measure the viability of A549 cells. The cells were seeded into 96‐well plates at a density of 5 × 10^3^ cells/well and cultured in an incubator at 37°C with 5% CO_2_. MTT (20 μL) solution was then added into each well to measure cell viability and incubated for another four hours. The media was then removed and 150 μL DMSO was added into the wells. The cell viability was calculated by measuring the optical density (OD) at 490 nm.

### Xenograft assays in athymic nude mice

The male BALB/c nude mice (fourth‐week old) were purchased from Vital River Laboratory. A549 cells were infected with miR‐133a lentivirus or control lentivirus by spin infection for two hours, followed by incubation at 37°C for two hours. A549 cells (resuspended to 10^6^ cells/mL) transfected with miR‐133a mimic were added to culture medium. The cells were injected subcutaneously into the right flank of the nude mice. Mice were sacrificed 28 days after inoculation (five in each group). None of the mice died during this study. Tumor volume was detected every four days. The nude mice were euthanized and tumor tissues used for further analysis. The present study was performed in strict accordance with the recommendations of the Guide for the Care and Use of Laboratory Animals of the National Institutes of Health. All experimental procedures involving animals were approved by the Animal Care and Use Committee of Linyi Central Hospital (2014–11‐156). As a humane endpoint, mice were euthanized if they met any of the following conditions: (i) they showed loss of >20% of bodyweight; ii) when the tumor mass was >10% of bodyweight; (iii) when an increased respiratory rate and/or effort was observed; (iv) if there was tumor necrosis or ulceration; and (v) if the mice showed an inability to access food or water. The mice were sacrificed by cervical dislocation performed under 1% sodium pentobarbital anesthesia 70 mg/kg, and all efforts were made to minimize suffering.

### Luciferase reporter assays

The wild‐ or mutant‐type of pGL3‐LASP1 3′UTR vector was constructed by pGL3‐reporter luciferase vector. A549 cells were cotransfected with 50 nM miR‐133a mimic and vector were seeded in six‐well plates and incubated for 24 hours according to the manufacturer's instructions. Dual‐luciferase reporter assay kit (Promega, Madison, WI) was used to measure luciferase intensity by a microplate reader. The ratio of Renilla luciferase to Firefly luciferase was calculated for each well.

### Statistical analysis

Data are represented as mean ± SD from three independent experiments. The difference between different groups was compared by two‐tailed Student's *t*‐test or one‐way ANOVA followed by Scheffe's post‐hoc analysis. The overall survival was analyzed by Kaplan‐Meier survival analysis. SPSS 19.0 software was used, and *P* < 0.05 indicated a statistically significant difference.

## Results

### MiR‐133a was downregulated, while LASP1 was upregulated in NSCLC

**Figure 1:**
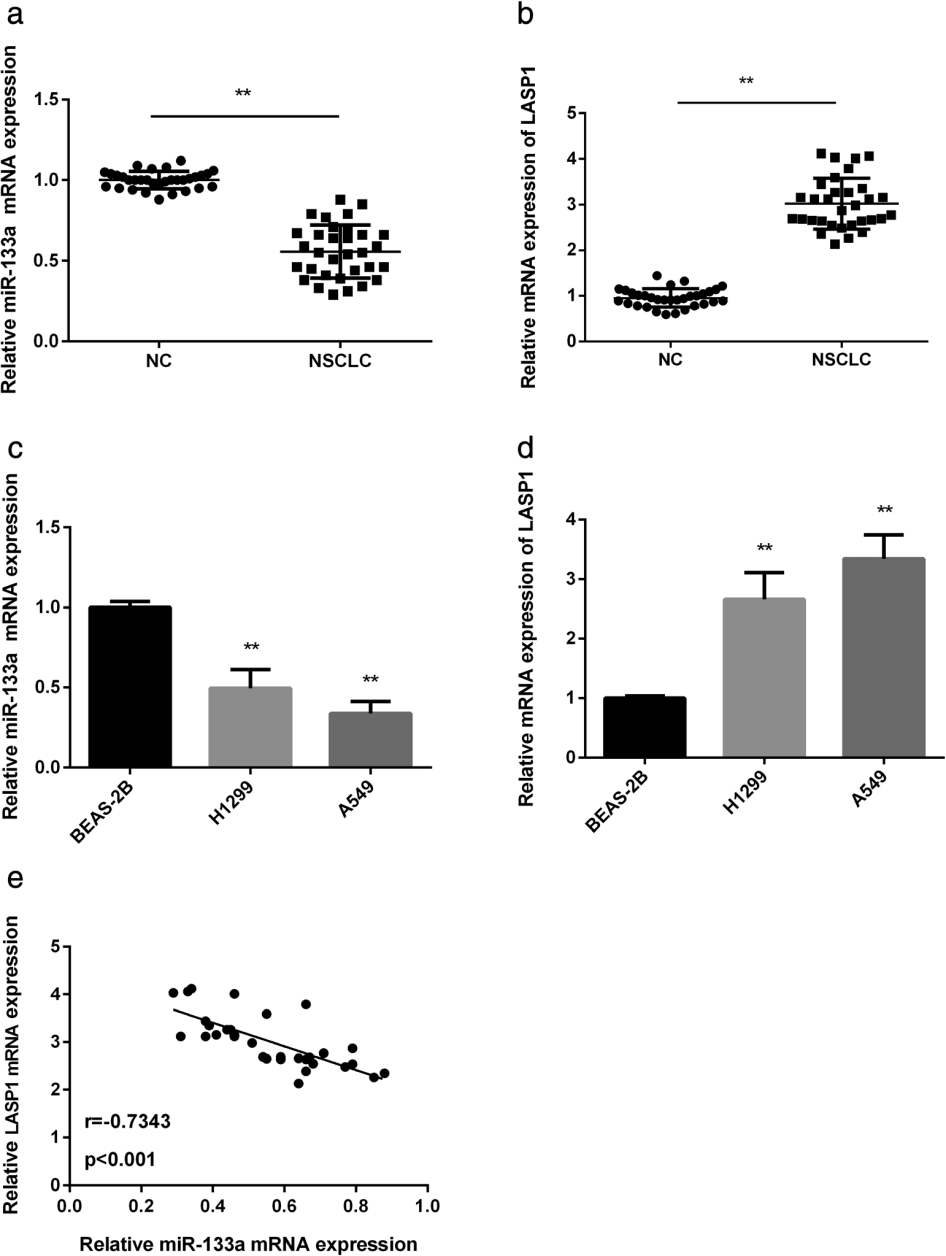
Low expression of miR‐133a and high expression of LASP1 were detected in NSCLC. (**a**) The expression of miR‐133a was examined in NSCLC tissues and normal tissues by qRT‐PCR (*n* = 32). (**b**) LASP1 expression was examined in NSCLC tissues and normal tissues by qRT‐PCR (*n* = 32). (**c**) The expression of miR‐133a was examined in NSCLC cell lines (H1299 and A549 cells) and normal lung epithelial cells (BEAS‐2B) by qRT‐PCR. (**d**) LASP1 expression was examined in NSCLC cell lines (H1299 and A549 cells) and normal lung epithelial cells (BEAS‐2B) by qRT‐PCR. (**e**) The relationship between miR‐133a and LASP1 expression was detected in NSCLC tissues (*n* = 32). **P < 0.01.

MiR‐133a and LASP1 expression levels in NSCLC tissues and cells were detected by RT‐PCR. Results showed that miR‐133a expression level was significantly downregulated in NSCLC tissues in contrast with adjacent noncancerous tissues (*P* < 0.01; Fig 1a). However, LASP1 expression level was upregulated in NSCLC tissues in contrast with adjacent noncancerous tissues (*P* < 0.01; Fig 1b). Moreover, as expected, miR‐133a expression was lower in H1299 and A549 cells than that in normal cells (*P* < 0.01; Fig 1c), while LASP1 expression was higher in both NSCLC cells than that in normal cells (*P* < 0.01; Fig 1d). Pearson correlation of the expression of miR‐133a and LASP1 in the tumor samples showed that they were negatively correlated (Fig 1d, r = −0.7343; *P* < 0.001).

### Overexpression of miR‐133a inhibited NSCLC cell viability

**Figure 2:**
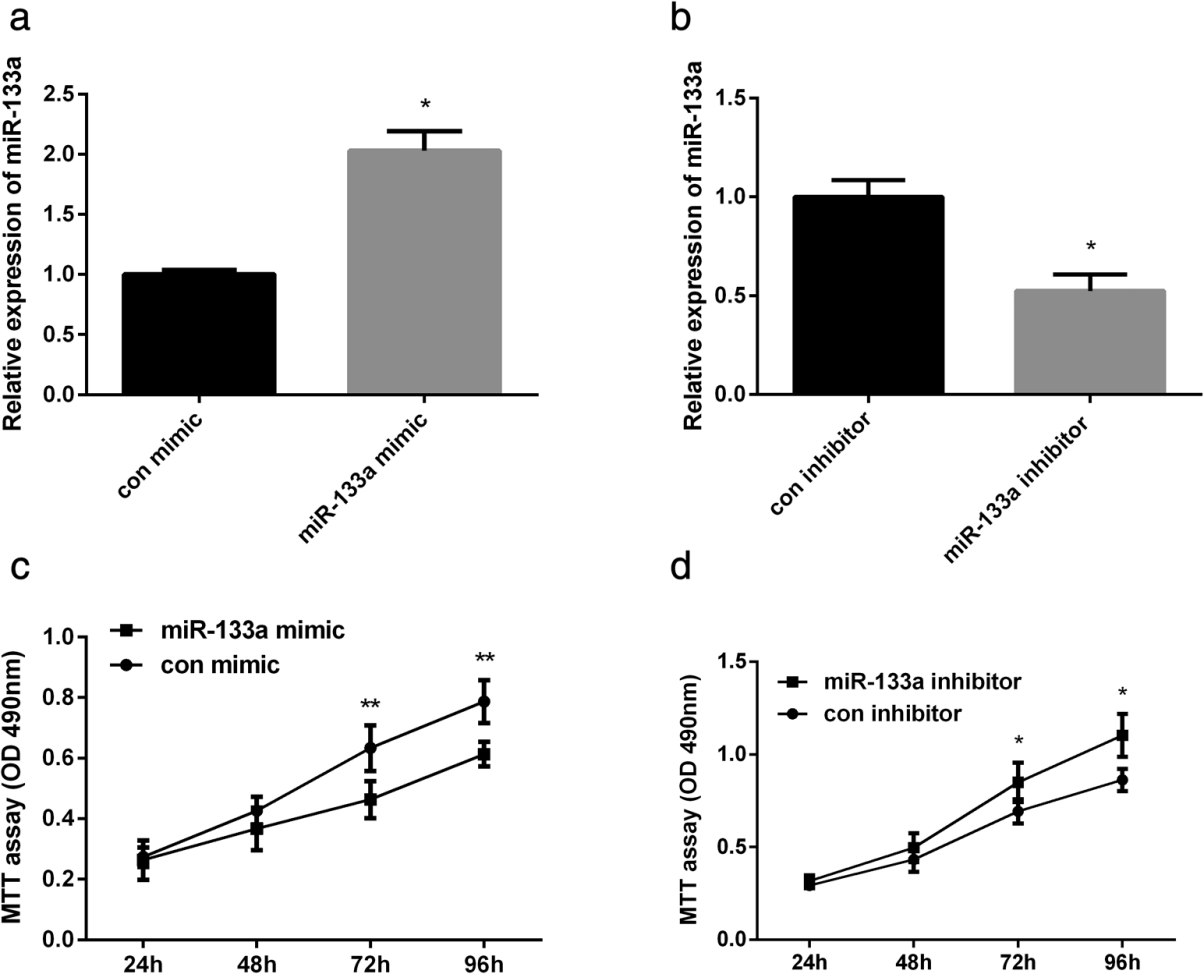
Inhibitory effect of miR‐133a on NSCLC cell proliferation was measured. (**a**) MiR‐133a expression was tested in A549 cells by qRT‐PCR after treatment with miR‐133a mimic. (**b**) MiR‐133a expression was tested in A549 cells by qRT‐PCR after treatment with miR‐133a inhibitor. (**c**) A549 cell viability was detected by MTT assay after treatment with miR‐133a mimic. (**d**) A549 cell viability was detected by MTT assay after treatment with miR‐133a inhibitor. *P < 0.05, **P < 0.01.

MiR‐133a mimic was successfully transfected into A549 cells to increase miR‐133a expression (*P* < 0.05; Fig 2a) and miR‐133a inhibitor was successfully transfected into A549 cells to decrease miR‐133a expression (*P* < 0.05; Fig 2b). MTT results showed that increasing miR‐133a expression by miR‐133a mimic could inhibit the viability of A549 cells, whereas suppression of miR‐133a expression by miR‐133a inhibitor could promote A549 cell viability (*P* < 0.01; *P* < 0.05; Fig 2c,d).

### LASP1 was a target of miR‐133a in NSCLC

**Figure 3:**
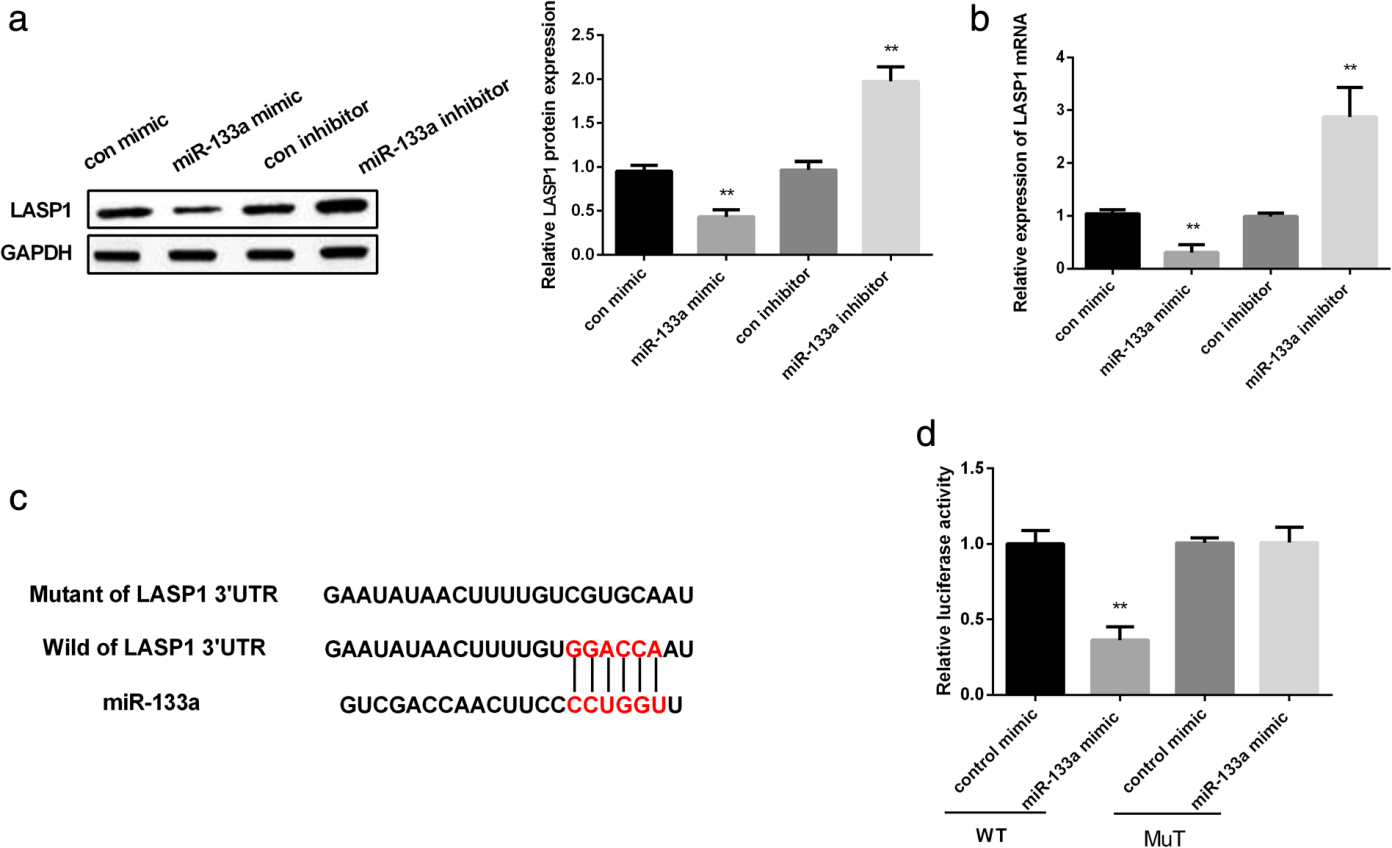
LASP1 was the target of miR‐133a in NSCLC. (**a**) LASP1 protein level was tested in A549 cells after treatment with miR‐133a mimic or inhibitor. (**b**) LASP1 mRNA expression was tested in A549 cells after treatment with miR‐133a mimic or inhibitor. (**c**) Prediction of the binding sites of miR‐133a with LASP1. (**d**) Luciferase activities were measured in A549 cells after treatment with miR‐133a mimic and pGL3‐LASP1 3′UTR vector. ** *P* < 0.01.

To investigate the impact of miR‐133a on LASP1 expression in NSCLC cells, qRT‐PCR and western blot were used in this study. As shown in Figure 3a, LASP1 protein level was markedly decreased by miR‐133a mimic, while increased by miR‐133a inhibitor (*P* < 0.01). Moreover, RT‐PCR results showed the same effect of miR‐133a on LASP1 expression (*P* < 0.01; Fig 3b). TargetScan, miRanda, and miRBase were then used to measure whether LASP1 was one of the potential targets of miR‐133a, as shown in Figure 3c, and the 3′‐UTR of LASP1 and miR‐133a showed binding sites. A luciferase reporter assay was then carried out to further confirm this prediction, as shown in Figure 3d, and overexpression of miR‐133a significantly decreased LASP1 3′‐UTR activity in the wild‐type group, while there was no significant change in the mutant group (*P* < 0.01). These results indicated that LASP1 was a direct target of miR‐133a in NSCLC.

### MiR‐133a mimic inhibited EMT and TGF‐**β**/Smad3 signaling pathway in vitro

**Figure 4:**
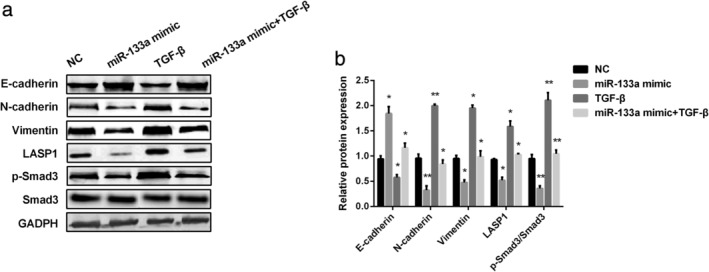
MiR‐133a inhibited EMT and TGF‐β/Smad3 pathway in vitro. (**a**) Western blot analysis of E‐cadherin, N‐cadherin, Vimentin, LASP1, Smad3, p‐Smad3 expression in A549 cells after treatment with miR‐133a mimic or stimulated by TGF‐β. (**b**) Quantification of E‐cadherin, N‐cadherin, vimentin, LASP1 and p‐Smad3/Smad3 expression in A549 cells after treatment with miR‐133a mimic or stimulated by TGF‐β.

LASP1 has been proven to regulate the EMT and TGF‐β signaling pathway.^16^ Here, we investigated whether miR‐133a regulated the EMT and TGF‐β signaling pathway via LASP1. E‐cadherin, N‐cadherin, vimentin, and Smad3, p‐Smad3 protein levels were examined by western blot in A549 cells transfected with miR‐133a mimic, TGF‐β or both miR‐133a and TGF‐β, respectively. As shown in Figure 4a,b, miR‐133a mimic increased E‐cadherin expression, while TGF‐β decreased its expression. However, miR‐133a mimic inhibited N‐cadherin and vimentin expression, while stimulating TGF‐β increased their expression. In addition, LASP1 was declined by miR‐133a mimic whereas increased by TGF‐β stimulation. p‐Smad3 expression level was declined by increasing miR‐133a and was raised by stimulating TGF‐β. However, TGF‐β stimulation could reverse miR‐133a effect on EMT and TGF‐β/Smad3 pathway in A549 cells, suggesting that TGF‐β/Smad3 pathway took part in NSCLC progression regulated by miR‐133a (*P* < 0.05, *P* < 0.01). In general, we concluded that miR‐133a might suppress NSCLC proliferation via TGF‐β/Smad3 pathway by targeting LASP1.

### Overexpression of miR‐133a inhibited tumor growth

**Figure 5:**
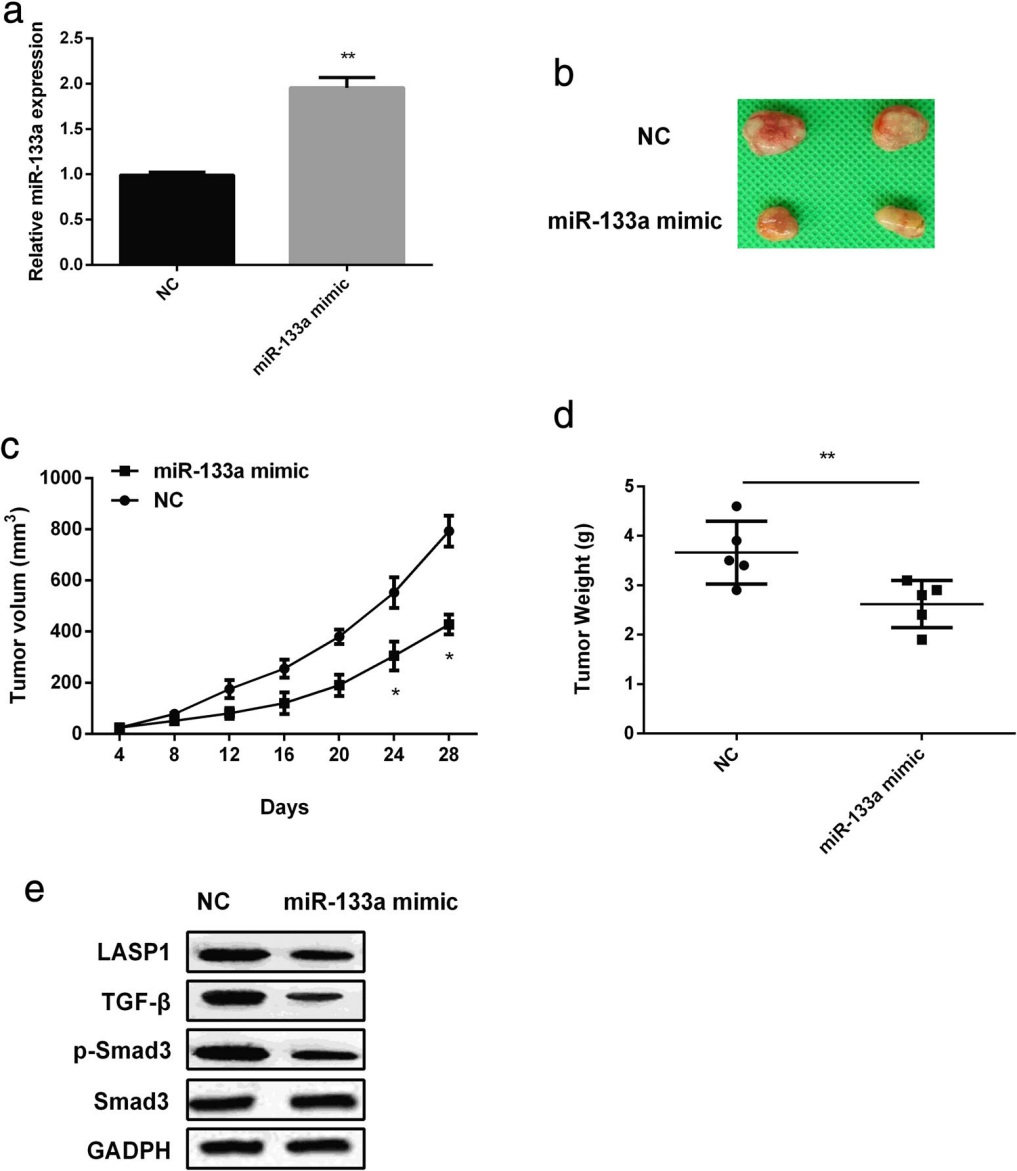
MiR‐133a inhibited tumor growth through TGF‐β/Smad3 pathway in vivo. (**a**) The mRNA expression of miR‐133a in xenografts tumors. (**b**) The tumorigenic ability of A549 cells after treatment with miR‐133a mimic. (**c**) The growth rate of tumors treated with miR‐133a mimic was slower than that in the control. (**d**) The weight of tumors treated with miR‐133a mimic was lighter than that in the control. (**e**) Western blot analysis of LASP1, TGF‐β, Smad3, p‐Smad3 expression in xenograft tumor tissues after treatment with miR‐133a mimic. *P < 0.05, **P < 0.01.

To evaluate the effect of miR‐133a on tumor growth in vivo, A549 cells transfected with miR‐133a mimic were injected subcutaneously into the nude mice. After injection for 28 days, the tumors were acquired. As shown in Figure 5a, the expression of miR‐133a was significantly increased in the tumor tissues of nude mice, and the tumor masses treated with miR‐133a mimic were smaller (Fig 5b). Moreover, compared with the control group, the tumor cell xenografts in the miR‐133a mimic group grew more slowly (*P* < 0.05; Fig 5c) and the tumors weighed less (*P* < 0.01; Fig 5d). We then investigated whether miR‐133a regulated the LASP1/TGF‐β/Smad3 signaling pathway in vivo, and western blot results revealed that miR‐133a restoration suppressed the expression of LASP1, TGF‐β and p‐Smad3 (Fig 5e), suggesting that miR‐133a might inhibit the tumor growth through TGF‐β/Smad3 signaling pathway by targeting LASP1.

## Discussion

MiRNAs play important roles in the regulation of gene expression, biological development, and carcinogenesis, suppressing transcriptional gene expression by combining with target messenger RNA (mRNA). Previous studies have shown that abnormal miRNA expression, which is involved in tumor development and metastasis, may be a perfect biomarker for the prognosis and diagnosis of cancers.^22^, ^23^, ^24^ In addition, miRNAs are continuously reported as being oncogenes or tumor suppressors. Since abnormal expression of miRNA is associated with NSCLC, research into the relationship between miRNAs and NSCLC has been promising. For example, miR‐21 has been reported to act as a novel biomarker for the prognosis and diagnosis of NSCLC.^25^ MiR‐539 has been reported to enhance the chemosensitivity to cisplatin in NSCLC cells.^26^ Jiang *et al*. reported that miR‐1258 as a tumor suppressor suppressed NSCLC progression.^27^ However, the effect of miR‐133a on NSCLC cell proliferation and tumor growth is not very clear. In our study, we found that miR‐133a was downregulated in NSCLC cells and tissues, and increasing miR‐133a inhibited NSCLC cell proliferation. Moreover, animal experiment results showed that overexpression of miR‐133a suppressed tumor growth in nude mice. Thus, we predicted that miR‐133a might act as a tumor suppressor in regulating NSCLC progression.

Based on biological information analysis, LASP1 has been predicted as the candidate target gene of miR‐133a. LASP1, a special focus adhesive protein, has been shown to play a part in some biological and pathological processes.^28^, ^29^ Previous studies have shown that LASP1 is involved in the progression of multiple tumors as an oncogene. LASP1 has been reported to be upregulated in colorectal cancer facilitating cell progression through the PI3K/AKT, TGF‐β/Smad3 signaling pathway.^16^, ^30^ Zhong *et al*. discovered that LASP1 regulated glioblastoma cell growth via the PI3K/AKT signaling pathway.^31^ In NSCLC, Zheng *et al*. determined that LASP1 facilitated cell proliferation and invasion.^19^ However, whether LASP1 is involved in the development of NSCLC regulated by miR‐133a has not yet been fully clarified. In our study, we found that LASP1 was a direct target of miR‐133a and involved in miR‐133a‐modulated NSCLC cell proliferation and tumor growth.

TGF‐β has been reported to regulate various immune response, adhesion, angiogenesis, tumors cell proliferation, migration and apoptosis.^32^ Jin *et al*. showed that TGF‐β inhibited tumor cell proliferation and promoted early tumor cell apoptosis while accelerating tumor angiogenesis, EMT and advanced cancer metastasis.^33^ It turns out that TGF‐β binds to its receptors, thus leading to phosphorylation of Smad3, which is a key factor in the TGF‐β signaling pathway. As the results indicate in this study, LASP1 and Smad3 expressions were upregulated by TGF‐β stimulation and downregulated in the miR‐133a mimic group. Moreover, E‐cadherin was inhibited by TGF‐β stimulation, while promoted by miR‐133a mimic. N‐cadherin and vimentin expression showed the opposite effect. Therefore, we determined that TGF‐β reversed the miR‐133a effect on EMT and Smad3 pathway in A549 cells, suggesting that miR‐133a achieved an antitumor effect by negatively regulating the TGF‐β/Smad3 pathway.

In conclusion, miR‐133a expression was downregulated while LASP1 was upregulated in NSCLC. Overexpression of miR‐133a inhibited NSCLC cell proliferation and tumor growth. LASP1 served as a target of miR‐133a in regulating NSCLC development and miR‐133a achieved an inhibitory effect on NSCLC by suppressing TGF‐β/Smad3 signaling pathway.

## Disclosure

The authors declare that they have no competing interests.
